# A three-dimensional model with two-body interactions for endothelial cells in angiogenesis

**DOI:** 10.1038/s41598-023-47911-1

**Published:** 2023-11-23

**Authors:** Kazuma Sakai, Tatsuya Hayashi, Yusuke Sakai, Jun Mada, Kazuo Tonami, Yasunobu Uchijima, Hiroki Kurihara, Tetsuji Tokihiro

**Affiliations:** 1 Graduate School of Mathematical Science, The University of Tokyo, 3-8-1, Komaba, Meguro-ku, Tokyo, 153-8914 Japan; 2 Faculty of Science and Engineering, Yamato University, 2-5-1, Katayama-cho, Suita, Osaka 564-0082 Japan; 3 Research and Development Initiative, Chuo University, 1-13-27, Kasuga, Bunkyo-ku, Tokyo, 112-8551 Japan; 4 Graduate School of Medicine, The University of Tokyo, 7-3-1, Hongo, Bunkyo-ku, Tokyo, 113-0033 Japan; 5 College of Industrial Technology, Nihon University, 1-2-1, Izumi-cho, Narashino, Chiba 275-8575 Japan; 6 Faculty of Engineering, Musashino University, 3-3-3 Ariake, Koto-ku, Tokyo, 135-8181 Japan

**Keywords:** Computational models, Applied mathematics

## Abstract

We introduce a three-dimensional mathematical model for the dynamics of vascular endothelial cells during sprouting angiogenesis. Angiogenesis is the biological process by which new blood vessels form from existing ones. It has been the subject of numerous theoretical models. These models have successfully replicated various aspects of angiogenesis. Recent studies using particle-based models have highlighted the significant influence of cell shape on network formation, with elongated cells contributing to the formation of branching structures. While most mathematical models are two-dimensional, we aim to investigate whether ellipsoids also form branch-like structures and how their shape affects the pattern. In our model, the shape of a vascular endothelial cell is represented as a spheroid, and a discrete dynamical system is constructed based on the simple assumption of two-body interactions. Numerical simulations demonstrate that our model reproduces the patterns of elongation and branching observed in the early stages of angiogenesis. We show that the pattern formation of the cell population is strongly dependent on the cell shape. Finally, we demonstrate that our current mathematical model reproduces the cell behaviours, specifically cell-mixing, observed in sprouts.

## Introduction

Angiogenesis, a biological phenomenon in which new vascular networks are constructed from existing blood vessels, plays a significant role in development, wound healing, menstruation, and pregnancy^1^. It is also implicated in the progression of pathological conditions such as malignant tumours and retinopathy^2^. Consequently, understanding the mechanisms of angiogenesis has become a crucial topic in modern medicine.

The general process of angiogenesis unfolds as follows. When cells surrounding an existing blood vessel become hypoxic or inflamed, they secrete vascular endothelial growth factor (VEGF) among other factors. This triggers the degradation of the underlying basement membrane, enabling endothelial cells to form new sprouts. Over time, these sprouts are lumenized, culminating in the formation of mature blood vessels. Particularly in the early stages of angiogenesis, endothelial cells exhibit complex behaviours such as overtaking each other, making U-turns, and intermixing, which are referred to as ’cell-mixing’^3,4^. Understanding endothelial cell behaviour during sprouting, such as ’cell-mixing’, is of considerable theoretical and experimental interest. It is also interesting to explore which properties of endothelial cells are associated with the vascular network.

Angiogenesis and vasculogenesis, the latter of wihch refers to the de novo formation of endothelial cells from mesodermal precursors, have inspired numerous mathematical models that focus on the formation of the initial embryonic vascular network^5–7^. For example, a mechanical model is based on the hypothesis that the extracellular matrix (ECM) is reorganised and the cellular networks are formed as a result of the traction forces exerted by the cells on the matrix and the elasticity of the matrix^8^. Cell movement is assumed to mimic a random walk with a bias towards areas of maximum strain, and cell locomotion is modelled as a diffusive movement with diffusion dependent on the local strain. According to these hypotheses, the model is described by nonlinear partial differential equations (PDEs). Another important factor in cell movement is the effect of chemoattraction. Before mesenchymal movement is activated, cells undergo a faster amoeboid-type migration driven by chemical factors such as Vascular Endothelial Growth Factor (VEGF). Mathematical models are proposed in which the cells are accelerated by gradients of chemoattractants released by the cells, diffuse and degrade in finite time^9,10^. They are described by PDEs for the fields of cell density (mass conservation law and the momentum balance equation) and chemotactic factor concentration (reaction diffusion equation).

Cell-based models have also been proposed for vascular network construction. Since cadherin-mediated cell-cell adhesion causes cell rearrangements that are analogous to the surface tension-driven dynamics of immiscible liquid droplets, Cellular Potts Models (CPM) are often used to represent cells as liquid droplets, whose area and perimeter are limited by mechanisms similar to surface tension and elastic compressibility^12,13^. Using CPM, it is shown that local inhibition of chemotaxis-induced pseudopod extensions by VE-cadherin induces the self-organization of endothelial cells into vascular-like networks^14^; including active cell motility with several assumptions such as each cell is capable of autonomous biased random motion, the directional bias is set by an internal polarity vector, statistical properties of the random streaming behaviour of endothelial monolayer cultures is studied^15^. By considering a set of rules for cell behaviour, including chemotaxis, haptotaxis, haptokinesis, and ECM-guided proliferation, a model with CPA and PDE for chemoattractant fields reproduces the formation of sprouts and branching vascular trees^16^. As another framework for cell shape, particle-based models, in which cells are represented by ellipses on a plane, have shown that ellipses with a large aspect ratio tend to form network patterns^17,18^.

In our previous research, we introduced a discrete dynamical model to describe the behaviour of endothelial cells during angiogenesis, based on results from in vitro studies^3,11^. Unlike the above models using PDEs and CPMs, we emphasised the importance of two-body interactions between cells and the deterministic movements caused by these interactions. Although the model did not include stochastic movements, chemoattractants, or the effects of ECM, we confirmed that deterministic interactions between two endothelial cells can lead to different outcomes, including cell-mixing, elongation, and branching. In this study, we extend our proposed two-dimensional model to ellipsoids in three dimensions to provide a basis for discussing lumen structures. When discussing cellular movements, such as cell-mixing within sprouts or network formation in the early stages of angiogenesis, it is reasonable to assume a two-dimensional system. However, we argue that a two-dimensional model falls short when considering three-dimensional structures, such as lumen formation^20^. Therefore, mathematical models that describe the movement of endothelial cells in a three-dimensional space are needed. Three-dimensional models related to angiogenesis have also been studied from several perspectives. An off-lattice, agent-based model has been developed for simulating vasculogenesis, with a focus on the formation of new blood vessels by endothelial progenitor cells^21^. The model categorises two types of endothelial cells as vessel elements within the network and tip cells located at the ends of vessels. It encompasses an array of forces, including mechanical and chemotactic forces, along with a distinctive ’persistence force’ for tip cells. Mathematical models, which use partial differential equations to link angiogenesis and three-dimensional tumour growth, have been proposed as a means of simulating tumour progression to enable chemotherapy evaluation^22,23^. In contrast to these models of angiogenesis, our mathematical framework offers a unique perspective and provides a different set of insights into the phenomenon.

In this paper, we introduce a dynamic system model that approximates endothelial cells as ellipsoids in a three-dimensional space. This model serves as an extension of our earlier two-dimensional framework, incorporating rotational effects resulting from two-body interactions and cell-cell contacts. Furthermore, we integrate new driving forces and rotational effects, transitioning from the longitudinal direction to the velocity direction, which have been shown to be crucial in the dynamics of two interacting cells^24^. Although our model is defined in a three-dimensional domain, it shares conceptual similarities with the model proposed by Palachanis et al.^17^, particularly in the approximation of cells as ellipsoids and the inclusion of both attractive and repulsive forces. The key difference with our model is that in Palachanis et al. the cell rotation rule is based on a Monte Carlo algorithm, whereas in the current model we have replaced this with rotational velocity. Numerical simulations confirm that the morphology of the cell population generated by our model depends on the shapes of the ellipsoids. We examine the underlying factors contributing to this dependency and identify model parameters that significantly impact the resultant patterns. To substantiate the robustness of our model, we explore the parameter range that yields configurations closely resembling vascular endothelial cell patterns. Finally, we performed three-dimensional culture experiments mimicking in vivo angiogenesis, which showed cell mixing phenomenon as observed in our previous two-dimensional experiments and models, validating our model for three-dimensional angiogenesis.

## Methods

### Contact of ellipsoids

**Figure 1:**
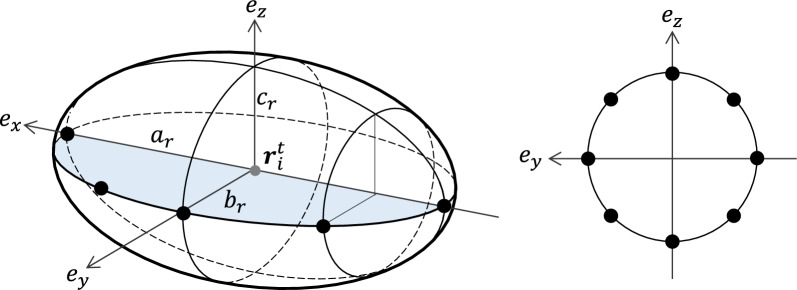
Sampling points for $K=4$ and $L=8$. (**a**) An ellipsoid with semi-axes $a_r$, $b_r$ and $c_r$. The axes $e_x$, $e_y$ and $e_z$ denote the major, middle and minor axis, respectively. The circles on the blue filled semi-ellipse represent the sampling points ${\varvec{x}}_i^t (\phi _k, \theta _0)$ ($k=0, 1, 2, 3, 4$). (**b**) The cross-section of (a) in the $e_y$-$e_z$ plane. The points on the ellipse represent the sampling points ${\varvec{x}}_i^t (\phi _2, \theta _l)$ ($l=0, 1, 2, \dots , 7$).

Let us consider the method to determine if two ellipsoids, representing cells in our model, are in collision. We assume that two ellipsoids are in contact if a point on the surface of one ellipsoid lies within the volume of the other ellipsoid. A point ${\varvec{x}}_i^t(\phi , \theta ) \in \mathbb {R}^3$ on an ellipsoid corresponding to the *i*th cell (cell-*i*) at time *t* is represented by the following parametric equation:

1 $$\begin{aligned} {\varvec{x}}_i^t (\phi , \theta ) = {\varvec{r}}_i^t + R_i^t \left( \begin{array}{ccc} a_r &{} 0 &{} 0 \\ 0 &{} b_r&{} 0 \\ 0 &{} 0 &{} c_r \end{array} \right) \left( \begin{array}{c} \cos \phi \\ \sin \phi \cos \theta \\ \sin \phi \sin \theta \end{array} \right) , \quad (0 \le \phi \le \pi ,\,\,0 \le \theta \le 2\pi ), \end{aligned}$$

where ${\varvec{r}}_i^{t} \in \mathbb {R}^3$ is the position of cell-*i* at time $t \in \mathbb {Z}_{\ge 0}$, $R_i^t \in SO(3)$ is the rotation matrix, and $a_r$, $b_r$, $c_r$ are positive real numbers ($c_r \le b_r \le a_r$). For $K ,L\in \mathbb {N}$, sampling points are generated by dividing the two angles given in equation (1) as $\phi =\phi _k = \pi k /K,\, \,\theta =\theta _l = 2\pi l/L\,\,(k\in \{0,1,\ldots ,K\},\,l\in \{0,1,\ldots ,L-1\})$ (Fig. 1). When sampling points ${\varvec{x}}_i^t (\phi _k, \theta _l)$ on the ellipsoid of cell-*i* are inside the ellipsoid of cell-*j*, the following inequality holds:

2 $$\begin{aligned} B_j^t({\varvec{x}}_i^t (\phi _k, \theta _l))^T \left( \begin{array}{ccc} 1/a_r^2 &{} 0 &{} 0 \\ 0 &{} 1/b_r^2 &{} 0 \\ 0 &{} 0 &{} 1/c_r^2 \end{array} \right) B_j^t({\varvec{x}}_i^t (\phi _k, \theta _l)) \le 1, \end{aligned}$$

where $B_j^t({\varvec{x}}):= (A_j^t)^T ({\varvec{x}} - {\varvec{r}}_j^{t})$. If the sampling points ${\varvec{x}}_i^t (\phi _k, \theta _l)$ of cell-*i* and cell-*j* satisfy inequality (Eq. 2) at time *t*, we say that “cell-*i* and cell-*j* are in contact”. In general, it is not easy to determine whether two ellipsoids interact with each other by solving two ellipsoid equations. However, it is relatively straightforward to confirm whether a point is inside an ellipsoid.

**Figure 2:**
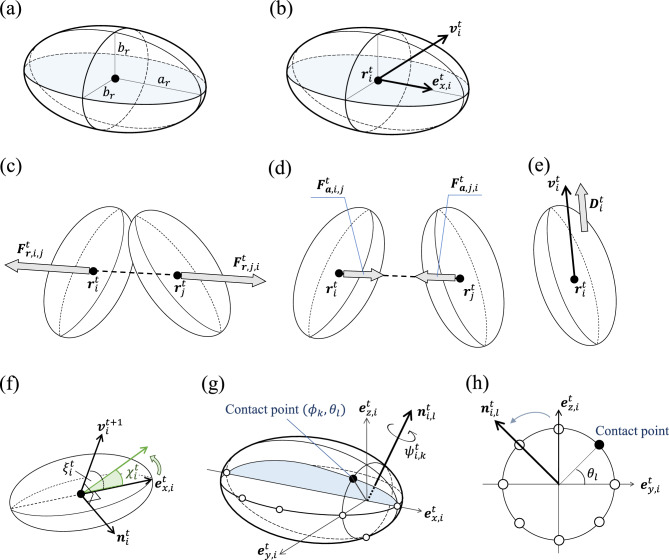
Schematic diagram of the proposed model. (**a**) A cell is represented as a spheroid with the semi-major axis $a_r$ and the semi-minor axis $b_r$. (**b**) The external force acts on the center ${\varvec{r}}_i^{t}$, and the velocity ${\varvec{v}}_i^{t}$ does not necessarily coincide with the direction of the major-axis ${\varvec{e}}_{x,i}^{t}$. (**c**–**e**) Forces acting on a cell: (**c**) repulsive force, (**d**) attractive force, (**e**) driving force. (**f**–**h**) Rotational forces. (**f**) Rotation toward the moving direction. The vector ${\varvec{e}}_{x, i}^{t}$ is rotated by $\chi _i^t$ around ${\varvec{n}}_{i}^{t}$. The vector ${\varvec{n}}_{i}^{t}$ is perpendicular to both ${\varvec{v}}_{i}^{t+1}$ and ${\varvec{e}}_{x, i}^{t}$. The angle $\chi _i^t$ is determined by Eq. (10). (**g**) Rotation due to cell-to-cell contact when $K=4$ and $L=8$. The black point is a contact point on the *l*-th semi ellipse filled in blue. The axis ${\varvec{e}}_{y,i}^t$ is perpendicular to both ${\varvec{e}}_{x,i}^t$ and ${\varvec{e}}_{z,i}^t$. The vector ${\varvec{e}}_{x, i}^{t}$ is rotated by $\psi _{i, k}^t$ around ${\varvec{n}}_{i,l}^{t}$. The angle $\psi _{i, k}^t$ is determined by Eq. (14). (**h**) The cross-section of (**g**) in the ${\varvec{e}}_{y,i}^t$- ${\varvec{e}}_{z,i}^t$ plane.

### Discrete mathematical model for endothelial cells in three dimensions

In this section, we describe a three-dimensional mathematical model of vascular endothelial cell dynamics. For simplicity, we represent each cell as a spheroid (a rotational ellipsoid) with the semi-major axis $a_r$ and the semi-minor axis $b_r=c_r$ (Fig. 2a). The state of cell-*i* at time *t* is characterised by three parameters: the centre of the spheroid ${\varvec{r}}_i^{t} \in \mathbb {R}^3$, the velocity ${\varvec{v}}_i^{t} \in \mathbb {R}^3$, and the direction of the major axis (Fig. 2b). Since we consider a spheroid, its direction is determined from the direction of only one axis. The three-dimensional model of dynamics of cell-*i* is given as follows:

3a $$\begin{aligned} {\varvec{r}}_i^{t+1}&= {\varvec{r}}_i^{t} + {\varvec{v}}_i^{t} , \end{aligned}$$

3b $$\begin{aligned} {\varvec{v}}_i^{t+1}&= {\varvec{v}}_i^{t} - \gamma _{1}{\varvec{v}}_i^{t} + {\sum _{j \ne i}} {\varvec{F}}_{i,j}^{t} + {\varvec{D}}_{i}^{t}, \end{aligned}$$

3c $$\begin{aligned} {\varvec{e}}_{x, i}^{t+1}&= R_{i}^{t} \,{\varvec{e}}_{x, i}^{t}, \end{aligned}$$

where $\gamma _{1}\,(0< \gamma _{1} < 1)$ denotes the friction coefficient, ${\varvec{F}}_{i,j}^{t}$ represents the two-body interaction between cell-*i* and cell-$j(\ne i)$, and ${\varvec{e}}_{x, i}^{t} \in \mathbb {R}^3$ is the unit vector that indeicates the direction of the major axis of the spheroid. Equations (3a) and (3b) are the discretised versions of Newton’s equations of motion, and Eq. (3c) is the equation of rotation.

Following our previous work^19,25^, we incorporate the two-body interaction ${\varvec{F}}_{i,j}^{t}$, which consists of a repulsive force due to the excluded volume effect and an attractive force resulting from the contact of pseudopods between cells. Let ${\varvec{F}}_{r,i,j}^{t}$ and ${\varvec{F}}_{a,i,j}^{t}$ represent the repulsive and the attractive forces between cell-*i* and cell-*j*, respectively (Fig. 2c, d). The interaction ${\varvec{F}}_{i,j}^{t}$ is expressed as follows:

4 $$\begin{aligned} {\varvec{F}}_{i,j}^{t} = {\varvec{F}}_{r,i,j}^{t} + {\varvec{F}}_{a,i,j}^{t}. \end{aligned}$$

The repulsive force ${\varvec{F}}_{r,i,j}^{t}$ is given by

5 $$\begin{aligned} {\varvec{F}}_{r,i,j}^{t} = -f_r \dfrac{{\varvec{r}}_j^{t} - {\varvec{r}}_i^{t}}{\Vert {\varvec{r}}_j^{t} - {\varvec{r}}_i^{t}\Vert }, \end{aligned}$$

where $f_r>0$ denotes the strength of the repulsion (Fig. 2 (c)). When the two spheroids are not in contact, ${\varvec{F}}_{r,i,j}^{t}={\varvec{0}}$. The attractive force ${\varvec{F}}_{a,i,j}^{t}$ is given by

6 $$\begin{aligned} {\varvec{F}}_{a,i,j}^{t} = {\left\{ \begin{array}{ll} f_a \dfrac{{\varvec{r}}_j^{t} - {\varvec{r}}_i^{t}}{\Vert {\varvec{r}}_j^{t} - {\varvec{r}}_i^{t}\Vert } &{}(\Vert {\varvec{r}}_j^{t} - {\varvec{r}}_i^{t}\Vert \le R_a), \\ {\varvec{0}} \quad &{}(\Vert {\varvec{r}}_j^{t} - {\varvec{r}}_i^{t}\Vert > R_a), \end{array}\right. } \end{aligned}$$

where $f_a>0$ denotes the strength of the attraction, and $R_a$ represents the radius of the interaction domain of cell-*i* (Fig. 2d). We assume $f_r>f_a>0$ due to the excluded volume effect. In Eq. (3b), ${\varvec{D}}_{i}^{t}$ represents the driving force. This force propels the cells in a certain direction and is defined as follows:

7 $$\begin{aligned} {\varvec{D}}_{i}^{t} = {\left\{ \begin{array}{ll} d\dfrac{{\varvec{v}}_i^{t}}{\Vert {\varvec{v}}_i^{t}\Vert } \quad &{}( {\varvec{v}}_i^{t} \ne {\varvec{0}} \,\, \textrm{and} \,\, \exists j\ne i\,;\Vert {\varvec{r}}_j^{t} - {\varvec{r}}_i^{t}\Vert \le R_a), \\ {\varvec{0}} \quad &{}(\textrm{otherwise}), \end{array}\right. } \end{aligned}$$

where *d* is a positive constant (Fig. 2e). This force represents the effect of enhanced motility when cell-*i* is not stationary and is interacting with another cell.

Equation (3c) indicates that the vector ${\varvec{e}}_{x, i}^{t+1}$ is determined by the rotation matrix $R_{i}^{t}\in SO(3)$. We consider two effects on the rotation: the rotation due to cell-to-cell contact and the rotation toward the direction of movement. Let $P_{i}^{t}$ and $Q_{i}^{t}\in SO(3)$ be the rotation matrices for cell-to-cell contact and the direction of movement, respectively. The rotation matrix $R_{i}^{t}\in SO(3)$ is given by

8 $$\begin{aligned} R_{i}^{t} = Q_{i}^{t} P_{i}^{t}. \end{aligned}$$

For the rotation matrix $Q_{i}^{t}$, we adopt the following form:

9 $$\begin{aligned} Q_{i}^{t}&= {\left\{ \begin{array}{ll} \exp \left[ \chi _{i}^{t} S({\varvec{n}}_i^{t}) \right] \quad &{}( {\varvec{v}}_i^{t+1} \ne {\varvec{0}}), \\ I_{3} \quad &{}({\varvec{v}}_i^{t+1} = {\varvec{0}}) , \end{array}\right. } \end{aligned}$$

10 $$\begin{aligned} \chi _{i}^{t}&= \gamma _{2}\sin (2\xi _{i}^{t}), \end{aligned}$$

11 $$\begin{aligned} {\varvec{n}}_i^t&= ({\varvec{e}}_{x,i}^{t} \times {\varvec{v}}_i^{t+1} ) / \Vert {\varvec{e}}_{x,i}^{t} \times {\varvec{v}}_i^{t+1}\Vert , \end{aligned}$$

where $\gamma _2>0$ denotes the strength of rotation, $\xi _{i}^{t}$ denotes the angle between ${\varvec{e}}_{x, i}^{t}$ and ${\varvec{v}}_i^{t+1}$, and $I_3$ denotes the unit matrix of order 3 (Fig. 2f). For ${\varvec{n}} = (n_x\,n_y\,n_z)^T \in \mathbb {R}^3$, $S({\varvec{n}})$ is defined by

12 $$\begin{aligned} S({\varvec{n}}) := n_x \left( \begin{array}{ccc} 0 &{} 0 &{} 0 \\ 0 &{} 0 &{} -1 \\ 0 &{} 1 &{} 0 \end{array} \right) + n_y \left( \begin{array}{ccc} 0 &{} 0 &{} 1 \\ 0 &{} 0 &{} 0 \\ -1 &{} 0 &{} 0 \end{array} \right) + n_z \left( \begin{array}{ccc} 0 &{} -1 &{} 0 \\ 1 &{} 0 &{} 0 \\ 0 &{} 0 &{} 0 \end{array} \right) . \end{aligned}$$

When ${\varvec{v}}_i^{t+1}\ne {\varvec{0}}$, the matrix $Q_{i}^{t}$ rotates a vector by the angle $\chi _{i}^{t}$ around the unit vector ${\varvec{n}}_i^t$. If the sign of $\chi _{i}^{t}$ is positive (negative), $Q_{i}^{t}$ rotates a vector in the anticlockwise (clockwise) direction about the rotation axis ${\varvec{n}}_i^t$. Then, the matrix $P_{i}^{t}$, which represents the rotation caused by cell-to-cell contact, is determined by *k*, *l* (Fig. 2g, h). For each *l*, the rotation axis ${\varvec{n}}_{i,\,l}^t$ is defined as follows:

13 $$\begin{aligned} {\varvec{n}}_{i,\,l}^t = \exp \left[ \dfrac{2\pi l}{L} S({\varvec{e}}_{x, i}^{t}) \right] {\varvec{e}}_{z, i}^{t}, \quad (l=0,1,2,\ldots ,L-1) \end{aligned}$$

where ${\varvec{e}}_{z, i}^{t}$ is the unit vector from the center ${\varvec{r}}_i^{t}$ to the point corresponding to $(\phi ,\theta )=(\pi /2,\pi /2)$. For each *k*, the rotation angle $\psi _{i,\,k}^{t}$ is defined as follows.

14 $$\begin{aligned} \psi _{i,\,k}^{t} = -f_p \sin (2\phi _k), \quad (k=0,1,2,\ldots ,K) \end{aligned}$$

where $f_p>0$ denotes the strength of the rotation due to cell-to-cell contact. For sampling points $(\phi _k, \theta _l)$ on the spheroid of cell-*i* that are inside that of cell-*j*, we obtain the matrix $P_{i}^{t}$ by multiplying the matrix $\exp [ \psi _{i,\,k}^{t} \,S({\varvec{n}}_{i,\,l}^t)]$ as

15 $$\begin{aligned} \displaystyle P_{i}^{t} = \exp \left[ \sum _{k,l}\psi _{i,\,k}^{t} \,S({\varvec{n}}_{i,\,l}^t) \right] . \end{aligned}$$

Note that rotations in three dimensions are generally non-commutative. However, since the parameters for rotation, $\gamma _2$ and $f_p$, are set to $10^{-3}$ to $10^{-2}$ in our simulation, we assume that the effect of the order of rotation is very small.

### Settings in numerical simulation

We characterise the shape of a spheroid using oblateness, defined as $\chi :=1-b_r/a_r$. We assume that each spheroid is scaled such that $a_r b_r^2=1$, ensuring a constant volume. Consequently, we have $a_r = (1-f)^{-2/3}$ and $b_r = (1-f)^{-1/3}$. The baseline parameters in our simulations are $K=10$, $L=8$, $R_a=1.25a_r$, $\gamma _1=0.1$, $f_r=0.02$, $f_a=0.001$, $d=0.002$, $f_p=0.001$, $\gamma _2=0.01$. Comparing with observations in the experiments^3^, we estimate that one time step is approximately two minutes and the unit length is about 40 ${\upmu }$m.

We conduct two types of numerical simulations, Simulation A and Simulation B, to investigate the effect of cell shape on pattern formation. Let $N_t$ be the number of cells at time *t*. In Simulation A, we start no cells at $t=0$, and a single cell is introduced at the origin every 10 time steps (i.e., $N_t = \lceil t/10 \rceil$). The initial velocity of an introduced cell is zero, and its major axis direction is randomly selected. In Simulation B, we start $N_t=2500$ cells are randomly distributed within a cube area at $t=0$, and no additional cells are introduced. Their initial velocities are zero, and directions of their major axes are randomly set.

To evaluate patterns and alignment, box-counting dimension and a local order parameter are used. The method for estimating the box-counting dimension is described in the Supplementary Information under “Estimation of the box-counting dimension”. Local alignment of elongated cells has been shown to play a crucial role in network formation^17,26^. To quantify this alignment, we adopted the following local order parameter:

16 $$\begin{aligned} \tilde{O}(\varepsilon ):= \dfrac{1}{N_t} \sum _{i=1}^{N_t} \, | \cos \sigma _i(\varepsilon ) | \end{aligned}$$

where $\sigma _i(\varepsilon ) \in [0, \pi )$ denotes the angle formed between ${\varvec{e}}_{x,i}^t$ and the average direction of the long axis of cell-*j* in the $\varepsilon$-neighborhood of cell-*i*, $\overline{{\varvec{e}}_{x,i}^t}:= \sum _{j \ne i; \Vert {\varvec{r}}_j^t - {\varvec{r}}_i^t \Vert <\varepsilon } {\varvec{e}}_{x,j}^t$. The absolute value of the cosine is adopted to equate cells facing in opposite directions. This order parameter $\tilde{O}(\varepsilon )$ takes the value of 1 for perfectly aligned spheroids and 0.5 for spheroids with random orientation.

### 3D cell culture

MS-1 cells were trypsinized and mixed with 1.5 mg/mL collagen matrix (Cellmatrix Type I-A, Nitta Gelatin) at a concentration of $2.2\times 10^4$ cells/200 $\upmu$L. The mixture was inoculated at the $\phi$14mm cavity of glass plates (D11130H, Matsunami) and placed in a CO$_2$ incubator for 1 h to let the gel solidate. Then 2 mL of DMEM supplemented with 10% fetal bovine serum (Nissui) and 1/20,000 volume of Syto-16 Green Fluorescent Nucleic Acid Stain (Thermo) was added to the plate. After one day, the plates were placed in a confocal laser scanning microscope (FV-10i, Olympus) and time-lapse fluorescence and phase-contrast images were obtained every five minutes over 8 h with 10 $\times$ 0.4 NA air objective lens.

### Cell tracking

The images obtained every five minutes were stacked in movies (7 flops per second) and movement of the cells were analyzed by manually tracking nuclear positions stained by Syto-16 using the manual tracking plug-in equipped in the software ImageJ2 version 2.14.0/1.54 f.

## Results and discussion

### Cell shape is instrumental in formation of branch-like structures

**Figure 3:**
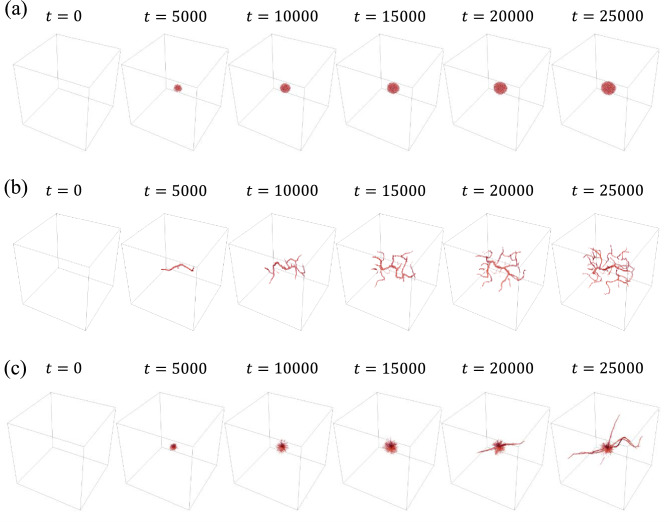
Snapshots of time evolution in Simulation A: (**a**) $\chi =0.3$, (**b**) $\chi =0.7$, (**c**) $\chi =0.9$.

The shape of cells, specifically their oblateness $\chi$, significantly influences pattern formation, as observed in both Simulation A and Simulation B. As depicted in Fig. 3, the time evolution from $t=0$ to $t=25,000$ in Simulation A demonstrates this effect. Round cells ($\chi =0.3$) tend to aggregate (Fig. 3a), whereas elongated cells ($\chi =0.7$) align by $t=5000$ and form a network structure by $t=25,000$ (Fig. 3b). More elongated cells ($\chi =0.9$) aggregate until $t=15,000$, after which branches grow from the cluster (Fig. 3c). Considering that a single cell is added every ten time steps, a total of 2500 cells are distributed by $t=25,000$.

**Figure 4:**
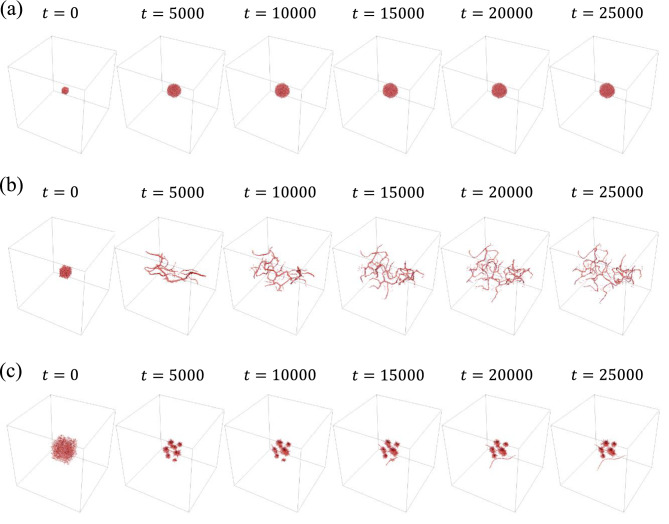
Snapshots of time evolution in Simulation B: (**a**) $\chi =0.3$, (**b**) $\chi =0.7$, (**c**) $\chi =0.9$.

Figure 4 shows the results of Simulation B for oblateness values of $\chi =0.3, 0.7, 0.9$. For an oblateness of $\chi =0.3$, cells aggregate near the center by $t=5000$, forming a large cluster without changing their positions. In contrast, elongated cells ($\chi =0.7$) form branch-like structures by $t=5000$. These branches then undergo repeated elongation and bending. For cells with a higher oblateness of $\chi =0.9$, clusters form up to $t=10,000$. After $t=15,000$, branches begin to grow from several clusters, while some cells continue to aggregate.

**Figure 5:**
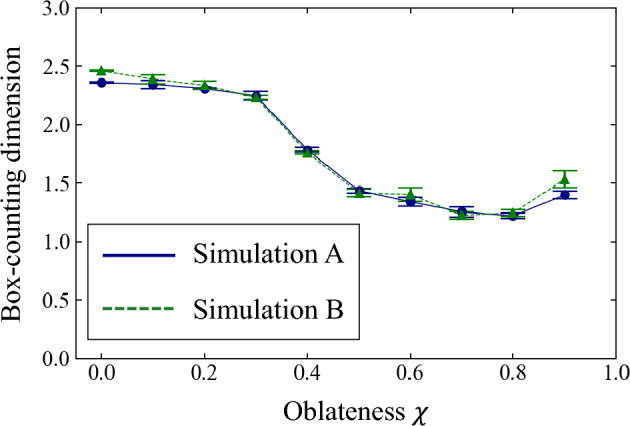
Box-counting dimension for Simulation A and B. The mean and 95% confidence interval are plotted after 10 iterations for each of oblateness from $\chi =0.0$ to $\chi =0.9$ in 0.1 increments. The parameters other than oblateness were $K=10,L=8,a_r b_r^2=1,R_a=1.25a_r,\gamma _1=0.1,f_r=0.02,f_a=0.001,d=0.002,f_p=0.001,\gamma _2=0.01$ for both simulations.

To assess the differences in pattern formation with respect to oblateness shown in Figs. 3 and 4, we compute the box-counting dimension. The box-counting dimension provides a measure of the complexity of the patterns formed by the cells, with lower values indicating simpler, more linear patterns and higher values indicating more complex, plane-filling patterns. Figure 5 shows the box-counting dimension computed for Simulation A and Simulation B at $t=25,000$. Oblateness is varied from $\chi =0.0$ to $\chi =0.9$ in 0.1 increments. Numerical simulation is performed 10 times for each oblateness. For both Simulation A and B, the box-counting dimension decreases monotonically from $\chi =0.0$ to $\chi =0.8$ and slightly increases at $\chi =0.9$. Notably, for $\chi =0.5\sim 0.8$, the box-counting dimension is close to 1.0. This result suggests one of the characteristics of the branch-like pattern shown in Figs. 3b and 4b. Since the box-counting dimensions for each oblateness are almost the same in the two simulations, Fig. 5 suggests that oblateness is crucial to the difference in pattern formation.

**Figure 6:**
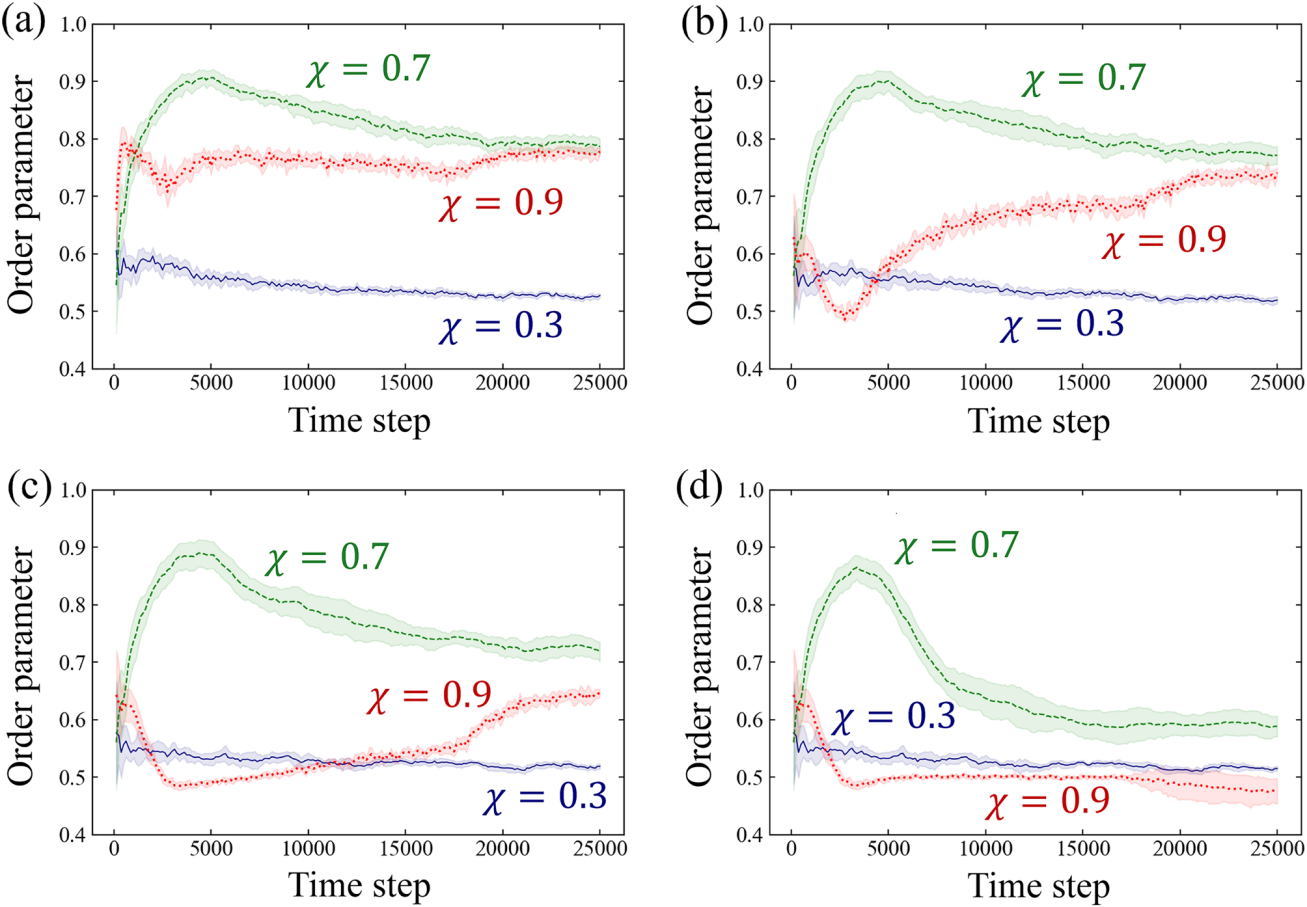
Temporal evolution of the order parameter for oblateness $\chi =0.3$, 0.7 and 0.9 in Simulation A: (**a**) $\varepsilon = 5$, (**b**) $\varepsilon = 10$, (**c**) $\varepsilon = 20$, (**d**) $\varepsilon \rightarrow \infty$. These plots show the average over 10 numerical runs and the bands show their 95% confidence intervals.

Figure 6 displays the time evolution of the order parameter for $\chi =0.3$, 0.7, and 0.9 in Simulation A. The results are presented for four radii $\varepsilon$: $\varepsilon =5$ to examine the orientation in the immediate vicinity of the cell, $\varepsilon =10,20$ to inspect the local orientation, and $\varepsilon \rightarrow \infty$ to investigate the global orientation. In the case of $\varepsilon \rightarrow \infty$, the order parameter is calculated for all cells at that time. For $\chi =0.3$, the order parameter remains low at all times, both locally and globally. Conversely, the order parameter for $\chi =0.7$ reaches a high peak before $t=5000$ both locally and globally, and then decays over time. Comparing with Fig. 3b, the branch structure is relatively linear at $t=5000$, while bending and bifurcation of the branches are observed afterward. For $\chi =0.9$, the order parameter in the vicinity of the cells ($\varepsilon =5$) remains stable after $t=5000$. For $\varepsilon =10, 20$, which includes more distant cells, the order parameter initially decreases, then tends to increase monotonically over time. For $\varepsilon \rightarrow \infty$, it initially decreases and remains relatively stable around $t=5000$, but the fluctuation increases around $t=20,000$. As shown in Fig. 3c, the branch starts elongating outward from the centre exactly around $t=20,000$, so the magnitude of this fluctuation is likely related to the number of cells in the elongating branch and the degree of freedom in the branch direction.

**Figure 7:**
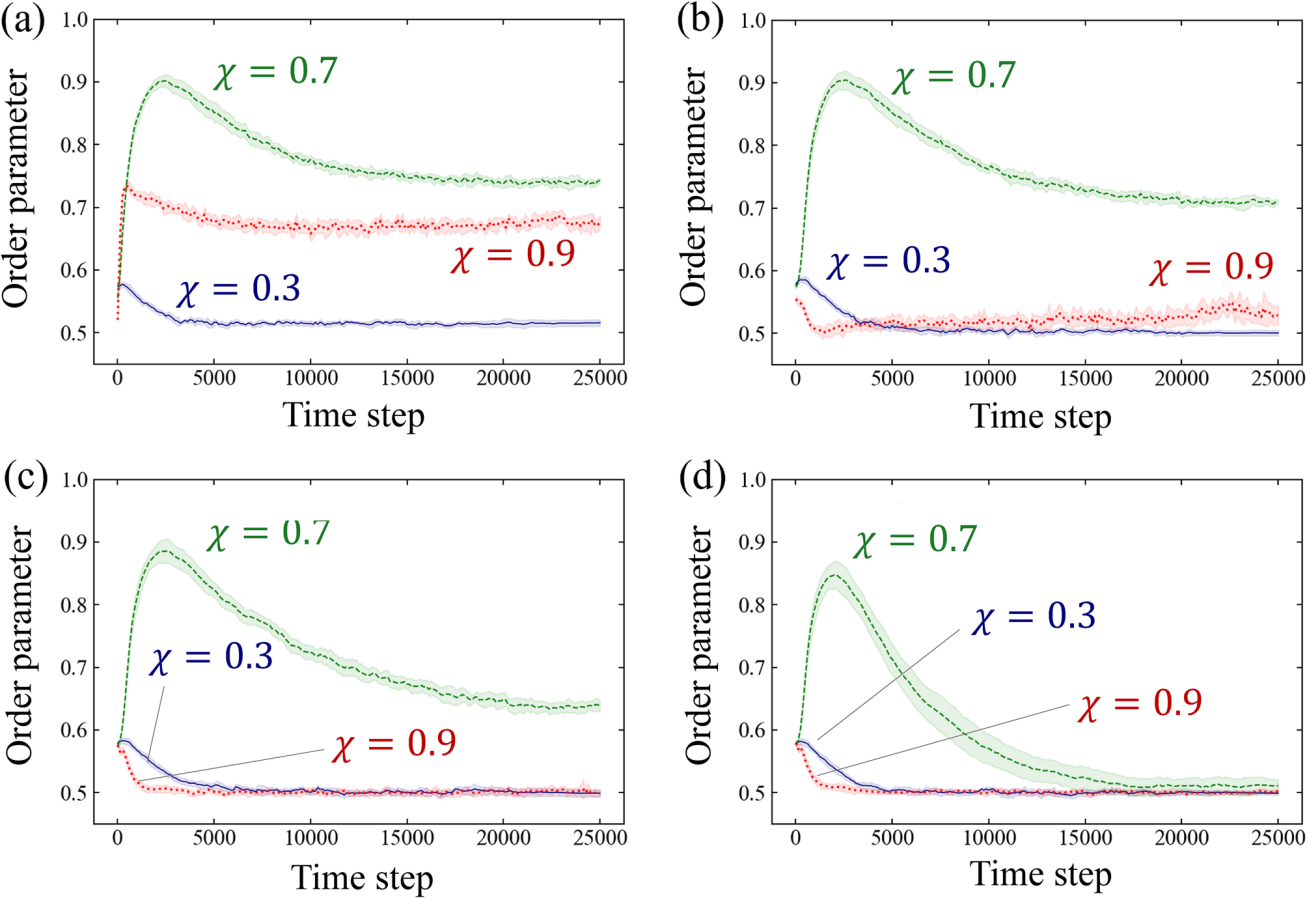
Temporal evolution of the order parameter for oblateness $\chi =0.3$, 0.7 and 0.9 in Simulation B: (**a**) $\varepsilon = 5$, (**b**) $\varepsilon = 10$, (**c**) $\varepsilon = 20$, (**d**) $\varepsilon \rightarrow \infty$. Each plot represents the average order parameter over 10 numerical runs, with the bands indicating their 95% confidence intervals.

We investigate the order parameter in Simulation B, as shown in Fig. 7. The results for $\chi =0.3$ and 0.7 are almost identical to those in Fig. 6. However, the results for $\chi =0.9$ show slight differences. For instance, in the case of $\varepsilon =10$ and 20, Fig. 6 shows that the order parameter decreases and then increases, while Fig.7 shows that it remains almost constant at a low value.

The analysis presented in Figs. 6 and 7 indicates that the orientation of cells is more aligned around the oblateness $\chi =0.7$ than in other cases. This difference in orientation leads to the variation in pattern formation shown in Figs. 3 and 4, particularly at $\chi =0.7$, where dendritic patterns resembling vascular structures are formed.

In our investigation of the effects of dimensionality on the order parameter, we compare the results of our three-dimensional model with those of a modified two-dimensional version. Specifically, we carry out simulations similar to Simulation B (Fig. S4). The findings are mostly similar between both dimensions. However, the order parameter between the models shows different behaviour when $\varepsilon \rightarrow \infty$. For example, the simulation in the three-dimensional model at $\chi =0.7$ displays an early-stage peak that is absent in the two-dimensional counterpart (refer to Fig. 7d and Fig. S4d). In the three-dimensional setting, initially positioned ellipsoids form one-dimensional structures with adjacent ellipsoids around $t=1250$ to $t=3750$ in Fig. S5, before they transition to a three-dimensional arrangement. This observation suggests an initial peak in the order parameter. Conversely, the two-dimensional model does not exhibit this early peak because the sprouts expand in all directions (Fig. S6).

In order to confirm the robustness of our proposed mathematical model, we examine the box-counting dimension when varying several parameters. We fix the oblateness at $\chi = 0.7$ and vary the parameters in the equations of motion, as well as the sampling point parameters *K* and *L* with respect to the ellipsoid. The ratio of the parameters related to repulsion and attraction is defined as $\lambda :=f_a/f_r$, and we investigate its relationship with the driving force *d*. For appropriate values of $\lambda$, no significant changes in the box-counting dimension are observed for $d=0.000$, 0.002, and 0.004 (Fig. S7a–c). The box-counting dimension remains largely unchanged for several combinations of the rotation parameters $f_p$ and $\gamma _2$ (Fig. S7d). In regard to the ellipsoid sampling point parameters *K* and *L*, we found that the box-counting dimension also does not vary significantly, as shown in Fig. S7e. Therefore, the current mathematical model is robust to these parameters with respect to the box-counting dimension.

### Biological validation of the current model

**Figure 8:**
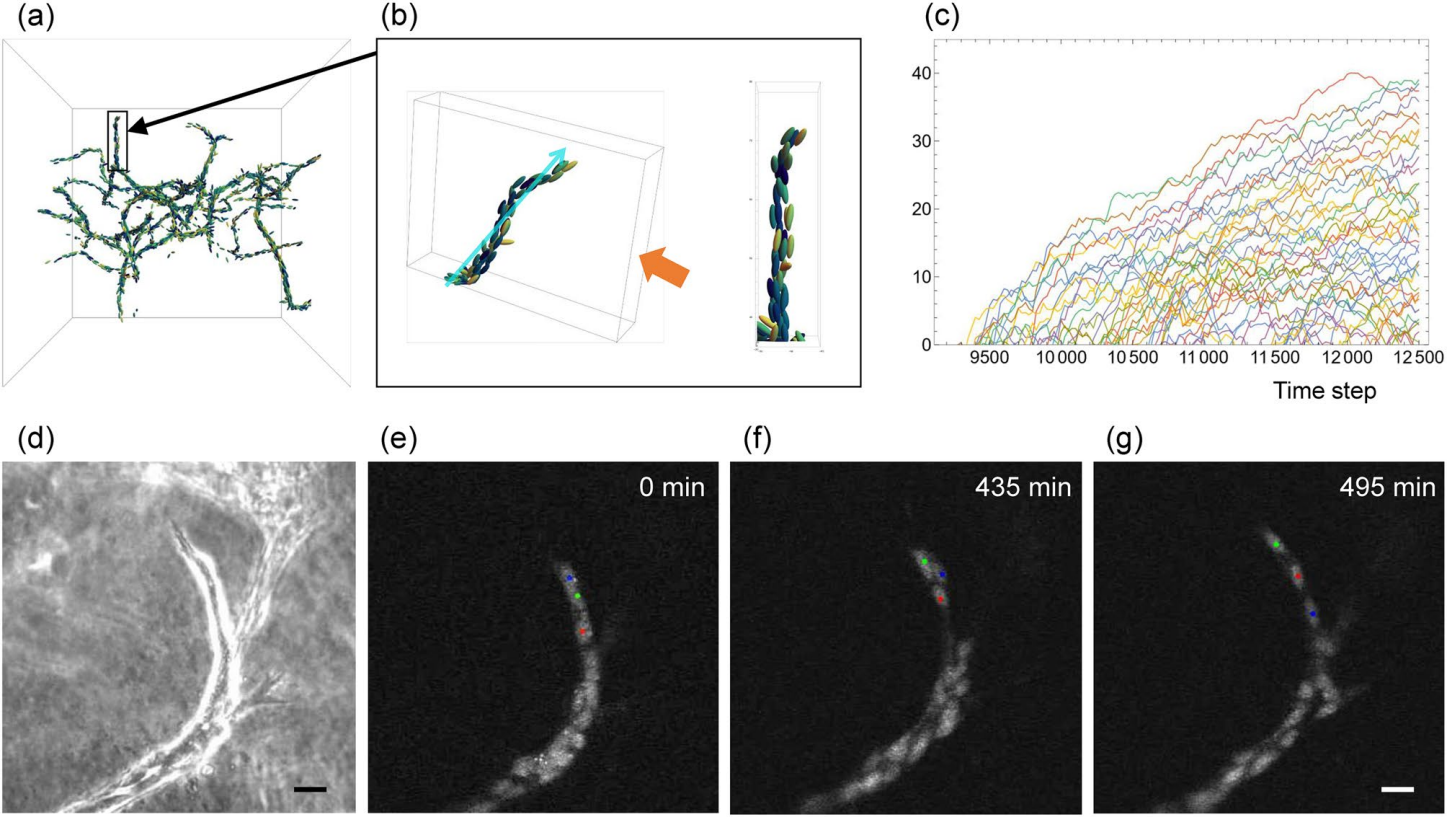
Comparison of the mathematical model with experimental observations. (**a,b**) Representative image of the simulation forming a branching network structure. The boxed branch in (**a**) is magnified in (**b**), where the right image is a longitudinal view of the left in the direction of the orange arrow. (**c**) Time evolution of cell positions in the branch in (**b**). Each line with different colour represents individual cell trajectory projected onto the blue arrow in (**b**). (**d**–**g**) Time-lapse images of endothelial cells during branch formation in three-dimensional culture. Phase-contrast (**d**) and fluorescent nuclear track (**e,f**) images are shown. Note that the positions of cells marked with different colours are changing within the branch. Scale bars, 20 $\upmu$m.

To assess the biological validity of the current model, we investigated cell behaviours within the simulation, particularly focusing on the formation of branch network structures. As seen in our previous two-dimensional models^18,19^, the current three-dimensional model successfully simulates sprouting angiogenesis with appropriate cell density along the branches (Fig. 8a, b). Additionally, we observed “cell mixing” phenomenon, which is characterized by endothelial cell behaviours involving forward and backward migration with frequent passing each other^3,4^ (Fig. 8c). To confirm the prevalence of this phenomenon in a three-dimensional cell environment, we conducted in vitro three-dimensional cultures of MS-1 cells, an immortalized endothelial cell line derived from murine pancreatic islet microvessels^27^. This culture system successfully reproduced angiogenic tube formation in collagen gel (Fig. 8d). Sequential nuclear tracking using the fluorescent dye Syto-16 revealed frequent cell mixing within the developing branches (Fig. 8e-g, Supplemental Movie S1). Collectively, these results support the validity of our current model for simulating three-dimensional angiogenic morphogenesis.

## Concluding remarks

We have presented a three-dimensional mathematical model of angiogenesis, extending our previous models^18,19^. In this model, each cell is approximated by a spheroid, and we have demonstrated that the shape of a spheroid significantly influences pattern formation. Branch-like patterns emerge when the oblateness is approximately 0.7. Elongated spheroids ($\chi =0.7$) can generate network structures under two different simulation conditions. We obtained qualitatively similar results when the parameters of interaction were varied, suggesting that these results are intrinsic features of our model. The proposed model successfully reproduces the pattern of elongation and branching observed in the initial stage of angiogenesis. We believe that this model can be further developed to simulate not only the initial stage of angiogenesis but also the formation of lumen structures.

### Supplementary Information

Supplementary Movie S1.

Supplementary Information.

## Data Availability

The datasets generated during and/or analyzed during the current study are available from the corresponding author on reasonable request. Customised scripts are available from https://github.com/RoastedGreenTeaaa/scripts-angiogenesis_3D.

## References

[1] Potente M, Gerhardt H, Carmeliet P (2011). Basic and therapeutic aspects of angiogenesis. Cell.

[2] Carmeliet P (2003). Angiogenesis in health and disease. Nat. Med..

[3] Arima S (2011). Angiogenic morphogenesis driven by dynamic and heterogeneous collective endothelial cell movement. Development.

[4] Jakobsson L (2010). Endothelial cells dynamically compete for the tip cell position during angiogenic sprouting. Nat. Cell Biol..

[5] Murray, J. D. *A Mechanical Theory of Vascular Network Formation*. 416–440 (Springer, 2003). 10.1007/0-387-22438-6_8.

[6] Czirok A, Little CD (2012). Pattern formation during vasculogenesis. Birth Defects Res. Part C Embryo Today Rev..

[7] Czirok A (2013). Endothelial cell motility, coordination and pattern formation during vasculogenesis. Wiley Interdiscip. Rev. Syst. Biol. Med..

[8] Manoussaki D, Lubkin S, Vemon R, Murray J (1996). A mechanical model for the formation of vascular networks in vitro. Acta Biotheor..

[9] Serini G (2003). Modeling the early stages of vascular network assembly. EMBO J..

[10] Tosin A, Ambrosi D, Preziosi L (2006). Mechanics and chemotaxis in the morphogenesis of vascular networks. Bull. Math. Biol..

[11] Sugihara K (2015). Autonomy and non-autonomy of angiogenic cell movements revealed by experiment-driven mathematical modeling. Cell Rep..

[12] Glazier JA, Graner F (1993). Simulation of the differential adhesion driven rearrangement of biological cells. Phys. Rev. E.

[13] Merks RM, Brodsky SV, Goligorksy MS, Newman SA, Glazier JA (2006). Cell elongation is key to in silico replication of in vitro vasculogenesis and subsequent remodeling. Dev. Biol..

[14] Merks RM, Perryn ED, Shirinifard A, Glazier JA (2008). Contact-inhibited chemotaxis in de novo and sprouting blood-vessel growth. PLoS Comput. Biol..

[15] Szabó A (2010). Collective cell motion in endothelial monolayers. Phys. Biol..

[16] Daub JT, Merks RM (2013). A cell-based model of extracellular-matrix-guided endothelial cell migration during angiogenesis. Bull. Math. Biol..

[17] Palachanis D, Szabó A, Merks RM (2015). Particle-based simulation of ellipse-shaped particle aggregation as a model for vascular network formation. Comput. Particle Mech..

[18] Hayashi T, Yura F, Mada J, Kurihara H, Tokihiro T (2022). Pattern formation of elliptic particles by two-body interactions: A model for dynamics of endothelial cells in angiogenesis. J. Theor. Biol..

[19] Matsuya K, Yura F, Mada J, Kurihara H, Tokihiro T (2016). A discrete mathematical model for angiogenesis. SIAM J. Appl. Math..

[20] Strilić B (2009). The molecular basis of vascular lumen formation in the developing mouse aorta. Dev. Cell.

[21] Perfahl H (2017). 3D hybrid modelling of vascular network formation. J. Theor. Biol..

[22] Tang L (2014). Computational modeling of 3D tumor growth and angiogenesis for chemotherapy evaluation. PloS one.

[23] Yanagisawa H, Sugimoto M, Miyashita T (2021). Mathematical simulation of tumour angiogenesis: Angiopoietin balance is a key factor in vessel growth and regression. Sci. Rep..

[24] Tonami, K. *et al.* Coordinated linear and rotational movements of endothelial cells compartmentalized by ve-cadherin drive angiogenic sprouting. *iScience* 107051 (2023). 10.1016/j.isci.2023.107051 PMC10329149 37426350

[25] Takubo N (2019). Cohesive and anisotropic vascular endothelial cell motility driving angiogenic morphogenesis. Sci. Rep..

[26] Palm MM, Merks RM (2013). Vascular networks due to dynamically arrested crystalline ordering of elongated cells. Phys. Rev. E.

[27] Arbiser, J. L. *et al.* Oncogenic h-ras stimulates tumor angiogenesis by two distinct pathways. *Proc. Natl. Acad. Sci.* **94**, 861–866 (1997). 10.1073/pnas.94.3.861 PMC19604 9023347

