# Supplementary material for: A three-dimensional model with two-body interactions for endothelial cells in angiogenesis
Source: Sci Rep. 2023 Nov 23;13:20549. doi: 10.1038/s41598-023-47911-1 (PMC10667370; doi:10.1038/s41598-023-47911-1)
Supplement: Supplementary file 2 — Supplementary Information. [file 41598_2023_47911_MOESM2_ESM.pdf]

Supplementary Information

A three-dimensional model with two-body interactions for endothelial cells in angiogenesis

Kazuma Sakai[1], Tatsuya Hayashi[2,3], Yusuke Sakai[4], Jun Mada[5], Kazuo Tonami[4], Yasunobu Uchijima[4], Hiroki Kurihara[4], and Tetsuji Tokihiro[1, 6]

1) Graduate School of Mathematical Sciences, The University of Tokyo, 3-8-1 Komaba, Meguro-ku, Tokyo, 153-8914, Japan.
2) Faculty of Science and Engineering, Yamato University, 2-5-1, Katayama-cho, Suita, Osaka, 564-0082, Japan.
3) Research and Development Initiative, Chuo University, 1-13-27, Kasuga Bunkyo-ku, Tokyo 112-8551, Japan.
4) Graduate School of Medicine, The University of Tokyo, 7-3-1, Hongo, Bunkyo-ku, Tokyo, 113-0033, Japan.
5) College of Industrial Technology, Nihon University, 1-2-1, Izumi-cho, Narashino, Chiba 275-8575, Japan.
6) Faculty of Engineering, Musashino University, 3-3-3 Ariake, Koto-ku, Tokyo 135-8181, Japan

Estimation of the box-counting dimension

For simplicity, we provide details of our methodology for estimating the box-counting dimension in the case of two dimensions. Consider a pattern S such as vascular branches and trees, represented by the solid lines in Fig. S1. This pattern is overlaid with a grid of boxes, each with a side length $\varepsilon > 0$. For each box size ε, let $N(\varepsilon)$ be the number of boxes that contain a portion of the pattern. For self-similar patterns, D_{BC} is called the box-counting dimension of S if $N(\varepsilon) \sim \varepsilon^{-D_{BC}}$ as $\varepsilon \to 0$.

In our simulations that involve vascular branches and trees, D_{BC} is numerically determined from the slope of the line when plotting $(\varepsilon, N(\varepsilon))$ on a log-log scale in the region corresponding to relevant scales. As shown in Fig. S2, we consider a scenario where 1000 ellipses are distributed on a plane, with configurations for $\chi = 0.0$, and $\chi = 0.7$. To compute the box-counting dimension for a given pattern, we count the number of boxes that contain the centre of an ellipse for each box size ε.

Figure S3 shows log-log plots of $(\varepsilon, N(\varepsilon))$ for the patterns in Fig. S2. Since we count the number of boxes containing the centre of an ellipse, $N(\varepsilon)$ does not exceed the number of cells for any given box size ε. If ε is sufficiently small, the number of boxes is approximately equal to the number of cells $N(\varepsilon) \sim 1000$. Our focus here is on vascular branches; therefore, we compute the line of best fit for a set of data where ε is larger than the cell scale, using least squares regression (Fig. S3). The slope of this regression line provides the estimated box-counting dimension for each pattern.

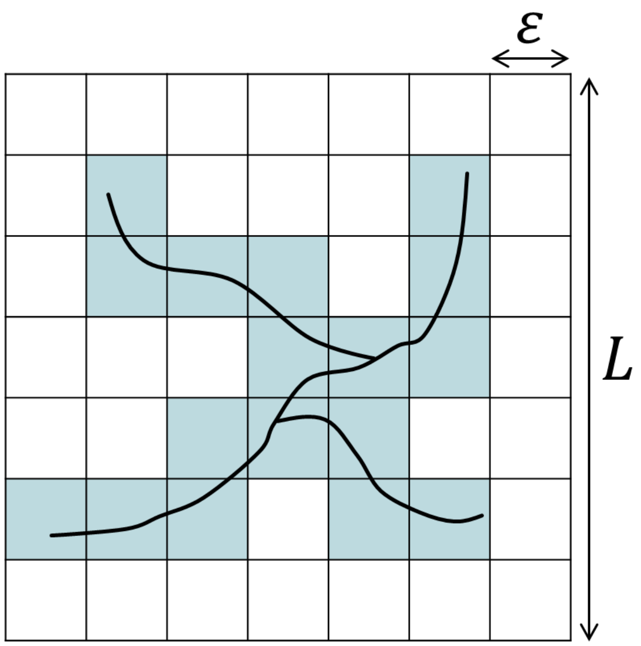

Figure S1. Covering a pattern with boxes of length ε. The filled squares show boxes that cover the pattern.

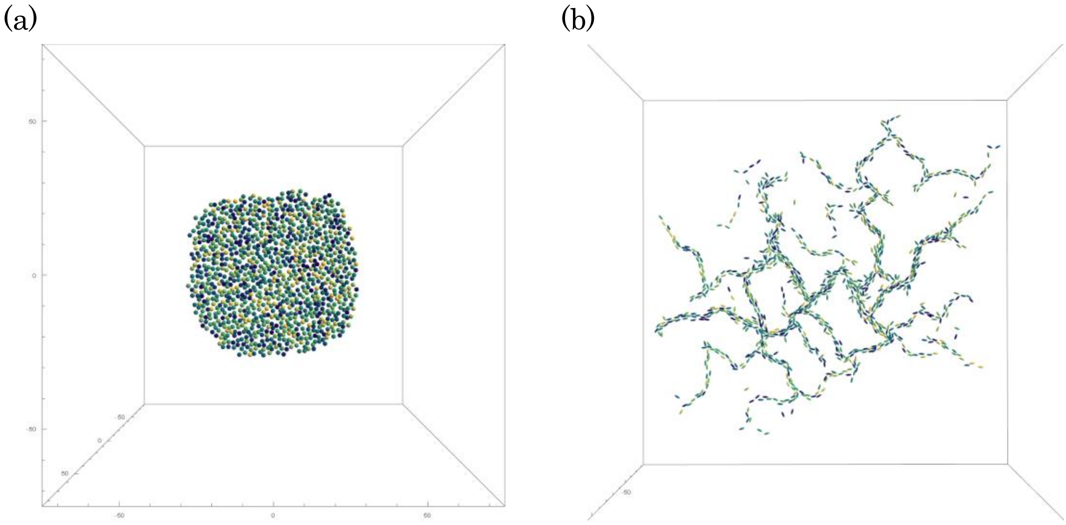

Figure S2. A snapshot from simulation for the distribution of 1000 ellipses with (a) $\chi = 0.0$, and (b) $\chi = 0.7$.

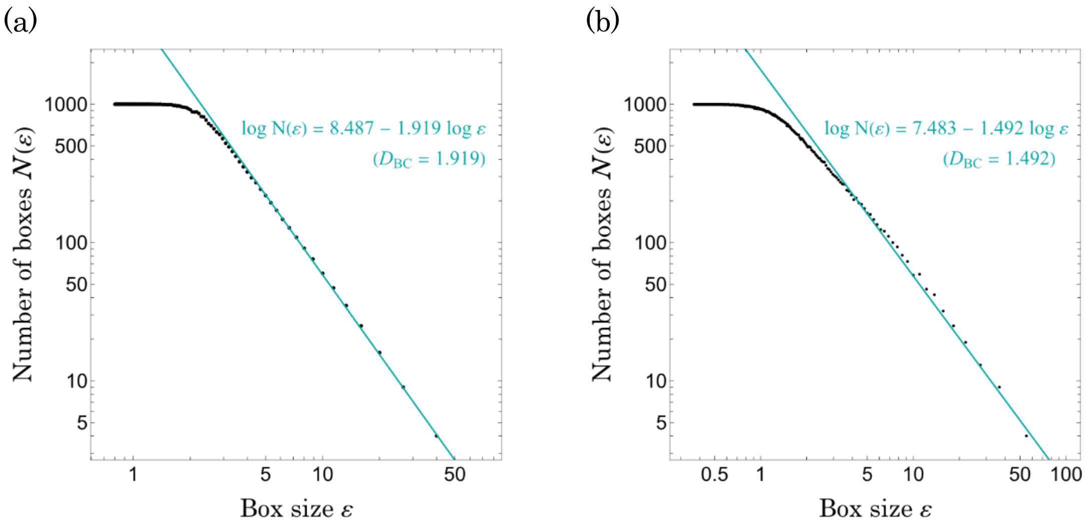

Figure S3. A plot of the number of boxes $N(\varepsilon)$ versus box size ε. The solid line shows a regression line that best fits the data before the plateau for (a) $\chi = 0.0$, and (b) $\chi = 0.7$.

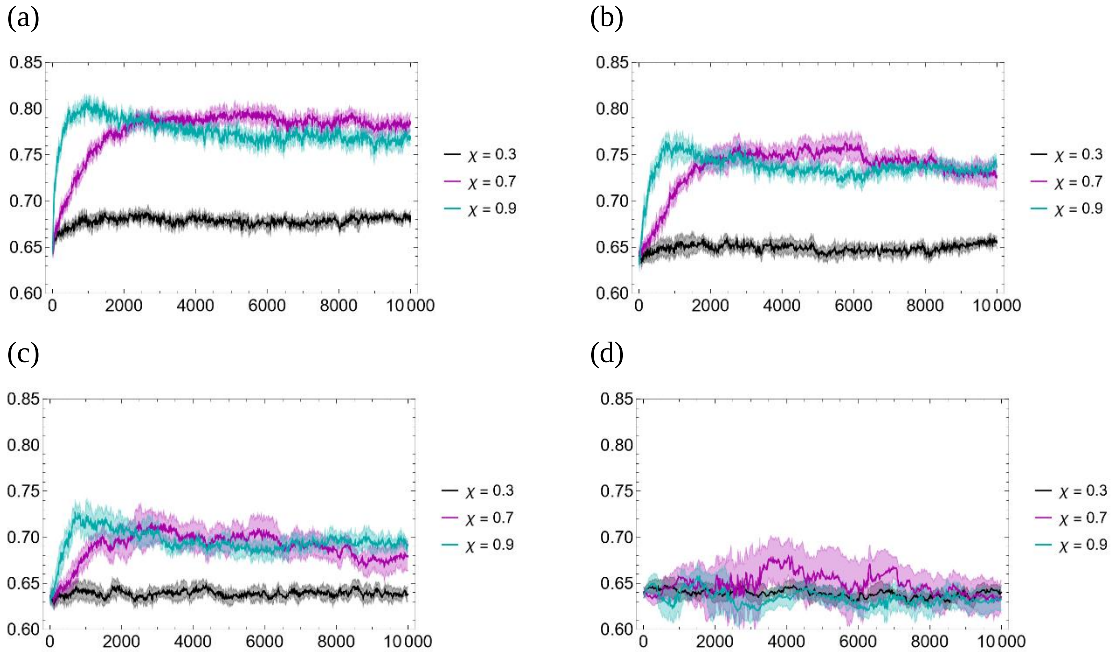

Figure S4. Temporal evolution of the order parameter for oblateness $\chi = 0.3$, 0.7 and 0.9 when (a) $\varepsilon = 5$, (b) $\varepsilon = 10$, (c) $\varepsilon = 20$, (d) $\varepsilon \to \infty$.

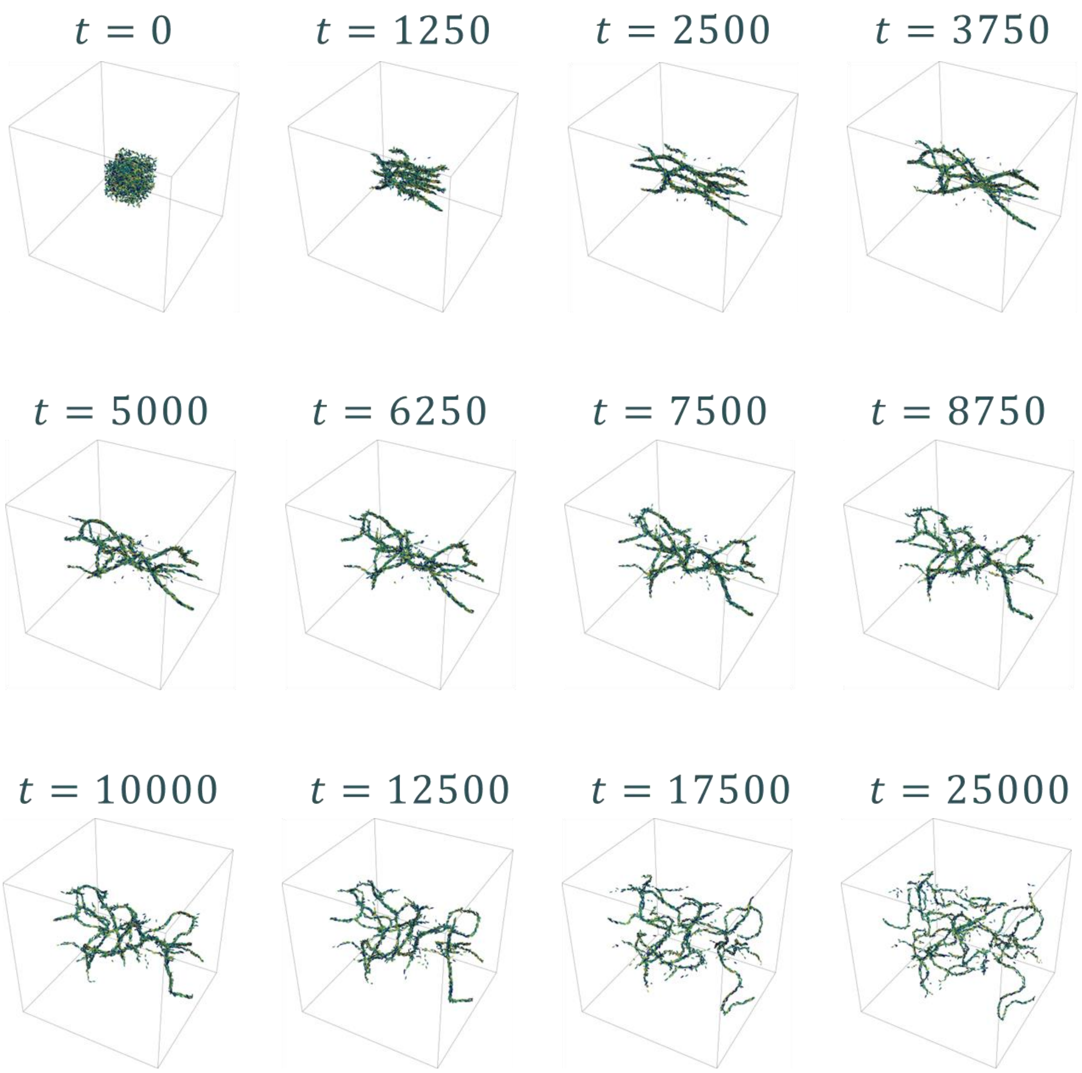

Figure S5. Snapshots of the time evolution by the three-dimensional model with $\chi = 0.7$.

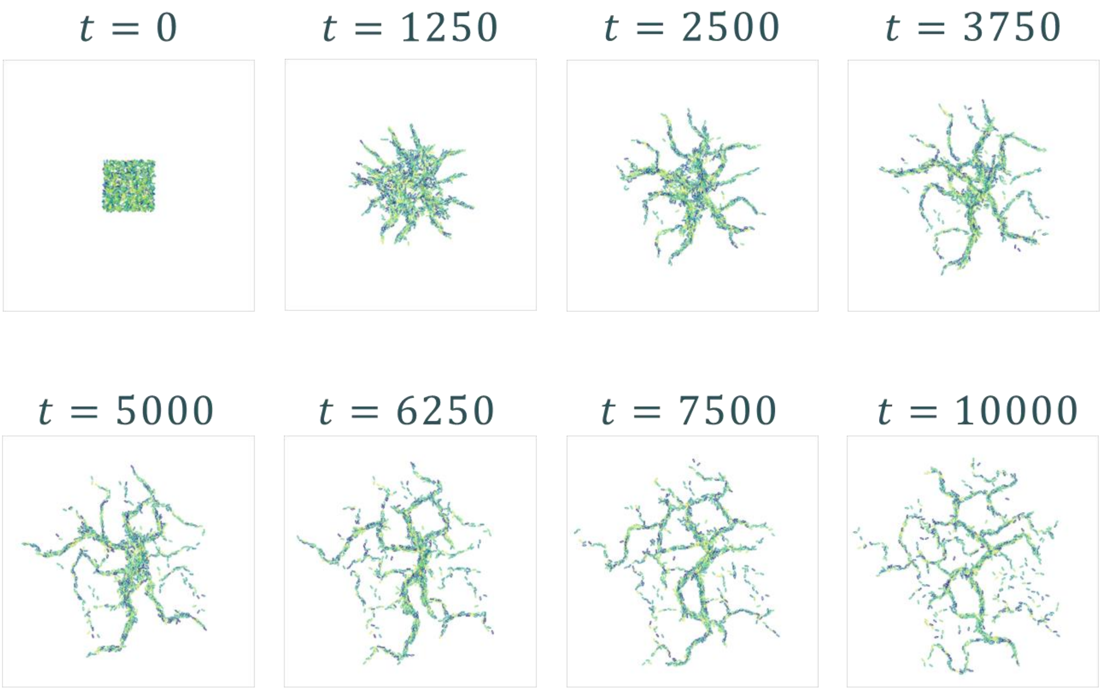

Figure S6. Snapshots of the time evolution by the two-dimensional model with $\chi = 0.7$.

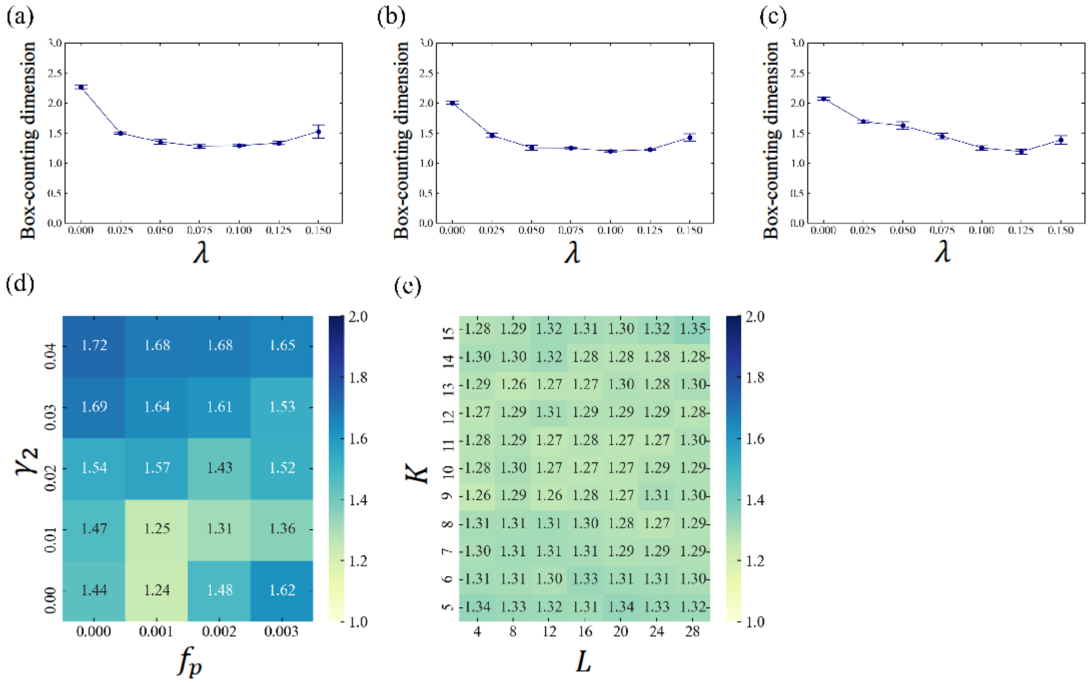

Figure S7. The impact of varying certain parameters on the pattern formation. Panels (a)-(c) show the results of Simulation A for oblateness $\chi = 0.7$, with the ratio $\lambda \coloneqq f_a/f_r$ varied for (a) $d = 0.000$, (b) $d = 0.002$, and (c) $d = 0.004$. The repulsion parameter is fixed at $f_r = 0.02$. Each plot represents the average of ten numerical simulations, with error bars indicating their 95% confidence intervals. Panel (d) represents a heatmap of the average box-counting dimension after ten numerical simulations for different f_p and γ_2. Panel (e) shows the dependence of the sampling points K and L in Simulation A for oblateness χ, with the heatmap displaying the average box-counting dimension after ten simulations for different K and L.

Movie S1. Dynamic behaviour of MS-1 cells in collagen gel. MS-1 cells were inoculated in collagen gel and images were taken every 5 minutes for over 8 hours as described in the experimental methods. Phase-contrast views (a), confocal fluorescent images of nuclei stained with Syto-16 dye (b), and confocal fluorescent images imposed with nuclear positions of representative cells as colour dots (blue, red, and green) in a forming branch. Scale bar, 20 μm.
